# Preparing criminal justice professionals to address new (post-)pandemic challenges as a result of criminals’ new *modi operandi*

**DOI:** 10.1007/s12027-022-00721-w

**Published:** 2023-01-10

**Authors:** Laviero Buono

**Affiliations:** Head of Section for European Criminal Law, ERA, Trier, Germany

The new European judicial training strategy for 2021–2024 states, page 4, under “Equipping practitioners to address new challenges” that: “The COVID-19 pandemic has altered criminals’ modi operandi, leading to a significant increase in offences involving cybercrime and online criminal activities. *Justice practitioners have to react to these changes. New training offers should be quickly organised and made available*” (emphasis added).^1^

Along these lines, also the Eurojust Annual Report 2020 states at page 53: “While the need for the digitalisation of the judiciary was clear long before the outbreak of the COVID-19 pandemic, the crisis was a catalyst in making ‘digital’ the default option in cross-border judicial cooperation to fight cross-border crime”.^2^

Finally, the Europol Internet Organised Crime Threat Assessment (IOCTA) 2020 dedicates an entire chapter at page 9 on how “Covid-19 demonstrates criminal opportunism” by stating that: “The difference with COVID-19 is that due to the physical restrictions enacted to halt the spread of the virus, with a subsequent increase in working from home and remote access to business resources, many individuals and businesses that may not have been as active online before the crisis became a lucrative target (for criminals)”.^3^

There is little doubt that COVID-19 did result in altering *modi operandi* of criminals. Offences related to cybercrime and online criminal activities increased significantly. Trade of illicit goods and services has moved even more to the Darknet, the number of phishing attempts, cases in online fraud, investment fraud, cyberattacks in the health area and trade in counterfeit medical products have increased. As children spend more time on-line, number of child sexual exploitation cases has also sharply risen in Europe. Internet related crimes have had more opportunities to hit and isolation has made people more vulnerable. To this extent, the protection of fundamental rights became even more important.

At operational level, judges, prosecutors and lawyers (as well as other criminal justice professionals) had to adjust their working methods to the new situation by organising online meetings, interrogations and hearings. To comply with safety measures during the COVID Pandemic, certain investigation methods, such as face-to-face interviews, interrogations, surveillance, arrests, house and on-site searches and audits could not be applied. Open source intelligence, use of social media, internet-related searches, cyber patrolling, videoconferencing involving vulnerable victims got even higher importance in investigations; the COVID Pandemic has proved that criminal legal practitioners need to improve knowledge in internet/online investigations in all crime areas.

Moreover, as a result of online investigations, almost all criminal courts are confronted with the question of whether or not electronic evidence presented in criminal proceedings is admissible. Rules governing the admissibility of electronic evidence vary in the legal framework of different Member States and are continuously challenged by the evolution of technological devices such as computers, mobile phones and digital cameras.

In recent years, important steps have been made at European level to develop an adequate legal framework to address the challenges posed by the gathering of e-evidence.

In April 2015, the European Union (EU) underlined possible solutions to allow for timely access to electronic evidence in its Communication ‘EU Agenda on Security’.^4^ The European Commission’s commitment was supported by the Council of the EU which adopted, on 9 June 2016, its Conclusions on ‘Improving criminal justice in the cyberspace’^5^ calling on the Commission to take actions to improve cooperation with service providers, make mutual legal assistance more efficient and propose solutions to the problems of determining and enforcing jurisdiction in cyberspace. As a consequence, an expert consultation process, including a detailed questionnaire, was launched in July 2017. The questionnaire revealed that there was no common approach to obtain cross-border access to electronic evidence for which each Member State had developed its own domestic practice. There is a diversity of approaches, mainly due to the lack of a common legal framework on obtaining e-evidence, which creates legal uncertainty and clear obstacles to cross-border investigations. As a result, a detailed technical document, although not adopted or endorsed by the European Commission, was presented in 2017. It was this document that laid down the foundation of the European Production Order. In fact, as a possible measure, the document stated: ‘A common framework across Member States could provide a basis for and recognise the legality of the current practices of direct cooperation, i.e. providing law enforcement and judicial authorities with the competence to make non-binding production requests for cross-border access to electronic evidence, and allowing service providers to disclose electronic evidence to foreign authorities on the basis of such a production request, without passing through local law enforcement or judicial authorities’.

As final preparatory paper, the European Commission issued an Impact Assessment in April 2018^6^ where it emerged clearly that in cross-border cases authorities have to rely on one of three channels: judicial cooperation between public authorities (often too slow), direct cooperation between public authorities and a service provider (often cumbersome and not transparent) and direct access to e-evidence (where legal frameworks remain fragmented). The document also indicated that although the European Investigation Order (EIO), in application since May 2017, covers the gathering and transfer of evidence between Member States and makes Mutual Legal Assistance (MLA) procedures faster, it is still considered insufficient, slow and therefore ineffective by national experts for accessing e-evidence in criminal investigations. Therefore, in the absence of EU intervention, the e-evidence gathering problem could only have been exacerbated by long time-consuming MLA requests and insufficient public-private cooperation between service providers and public authorities.

On 17 April 2018, the European Commission proposed new rules to better equip law enforcers and judicial authorities. As a matter of fact, the EIO and the MLA procedures will continue to exist, but there will be new avenues, or ‘fast tracks’ for the specific case of electronic evidence. The new legal framework that builds upon the provisions of the EIO, which effectively provides assistance between law enforcement and judicial authorities in different EU Member States, will complement it by creating a set of clear and coherent principles to enable requests by law enforcement and judicial authorities in one Member State to be made directly to a service provider in another Member State for the disclosure of data. This new set of rules consists of two proposals: 1) the Proposal for a Regulation on European Production and Preservation Orders for electronic evidence in criminal matters^7^ and 2) the Proposal for a Directive laying down harmonised rules on the appointment of legal representatives for the purpose of gathering evidence in criminal proceedings.^8^

In response to two recent European Commission Calls,^9^ ERA presented two multi-annual Projects which addressed various challenges that judges, prosecutors and lawyers in private practice working in the field of EU criminal justice will have to face for the years ahead. Some of these challenges (e-evidence, videoconferencing, wide use of open source intelligence tools, internet-related crimes, digital tech, etc.) are there to stay also in the “new normal” and well beyond the end of the Pandemic.

The first series, 2021–2022, provided in-depth training on e-evidence, an area of law where future well-coordinated training schemes are needed to provide to the largest possible number of EU legal practitioners the basic skills necessary to cope with the handling of digital evidence in courts. Six training events organised across the European Union face to face and online.^10^

The second series, 2022–2024, focuses on the (post)COVID challenges in criminal justice for EU legal practitioners. The seven face to face training seminars contemplated in this series (40 participants per seminar x 7 seminars = 280 participants), to be implemented in various EU cities (Bucharest, Dublin, Lisbon, Cracow, Barcelona, Thessaloniki and Tallinn) will be complemented by six video podcasts, 45 mins each, related to the core topics, which can also be used as stand-alone product (e-learning). Moreover, audio podcasts will be produced and made available for a wider audience. In MP3 format, audio podcasts can be enjoyed on-the-go on portable devices (mobile learning or m-learning). The creation of a dedicated project subsite on ERA’s website, including video and audio podcasts, documentation and speakers’ contributions will also be further project results: https://era-comm.eu/postcovid-challenges-criminal-justice/.

By participating to the seven training courses of this Project EU judges, prosecutors and lawyers in private practice will be provided with the “key skills” necessary to cope with all those criminal offenses which present a strong tech/internet component. The seven training courses will address the challenges when investigating, engaging in pre-trial preparation and presenting the case involving a variety of scientific evidence and/or internet sources. To this extent, the training activities will be in line with the work carried out by the European Union in the field of e-evidence. If eventually adopted, all seminars contemplated in the series will dedicate ample space to the presentation of the new 2018 EU legal instruments on the Preservation and Productions Orders and on direct cooperation with service providers.

The courses, all conducted in a “learning-by-doing” style which, through demos and simulations will offer an insight into what devices hold the most information and how sources such as PCs, tablets, smart phones, laptops and the Internet are investigated. As well as learning about the process, the participants will understand the results of investigations, their presentation/admissibility in court, gaining an understanding on what can and cannot be found in digital devices.

Moreover, the courses will introduce participants to the concepts and the world around the internet and its supporting tools for investigation/research. Dedicated modules will unravel internet search engine tools and make legal practitioners aware of the sources of evidence available to them in online investigations.

Undoubtedly, this Project will also contribute to the “knowledge of other legal systems” by involving experienced legal practitioners who will be able to compare national experiences and practices as practical experience to establish the admissibility, the authenticity and the validity of the electronic evidence is still very limited.

## Footnotes

1 Communication from the Commission to the European Parliament, the Council, the European Economic and Social Committee and the Committee of the Regions: Ensuring justice in the EU — a European judicial training strategy for 2021–202 (Brussels, 2.12.2020 COM(2020) 713 final).

2 Eurojust Annual Report 2020 – Criminal Justice Across Borders. Available at: https://www.eurojust.europa.eu/ar2020.

3 Europol Internet Organised Crime Threat Assessment (IOCTA) 2020. Available at: https://www.europol.europa.eu/publications-events/main-reports/internet-organised-crime-threat-assessment-iocta-2020.

4 Communication from the Commission to the European Parliament, the Council, the European Economic and Social Committee and the Committee of the Regions: The European Agenda on Security (Strasbourg, 28.4.2015 COM (2015) 185 final).

5 Council Conclusions on Improving criminal justice in the cyberspace (Luxembourg, 9 June 2016).

6 European Commission, Impact Assessment Report (Brussels, 17.4.2018 SWD(2018) 118 final).

7 Proposal for a Regulation of the European Parliament and the Council on the European Production and Preservation Orders for electronic evidence in criminal matters (Strasbourg, 17.4.2018 COM(2018) 225 final).

8 Proposal for a Directive of the European Parliament and of the Council laying down harmonised rules on the appointment of legal representatives for the purpose of gathering evidence in criminal proceedings (Strasbourg, 17.4.2018 COM(2018) 226 final).

9 European Commission Justice Programme Calls: Just-JTRA-EJTR-AG-2018 and JUST-2021-JTRA.

10 For a complete overview of the Project (objectives, partners, library, etc.) see the ad hoc subsite: https://era-comm.eu/e-evidence/.

